# Longitudinal assessments highlight long-term behavioural recovery in disorders of consciousness

**DOI:** 10.1093/braincomms/fcz017

**Published:** 2019-09-16

**Authors:** Corinne A Bareham, Judith Allanson, Neil Roberts, Peter J A Hutchinson, John D Pickard, David K Menon, Srivas Chennu

**Affiliations:** 1 Department of Clinical Neurosciences, University of Cambridge, Cambridge CB2 0QQ, UK; 2 Cambridge University Hospitals NHS Foundation Trust, Cambridge CB2 0QQ, UK; 3 Sawbridgeworth Medical Services, Jacobs & Gardens Neuro Centres, Sawbridgeworth CM21 0HH, UK; 4 Division of Anaesthesia, University of Cambridge, Cambridge CB2 0QQ, UK; 5 School of Computing, University of Kent, Chatham Maritime, ME4 4AG, UK

**Keywords:** Coma Recovery Scale-Revised, disorders of consciousness, arousal, natural history, longitudinal

## Abstract

Accurate diagnosis and prognosis of disorders of consciousness is complicated by the variability amongst patients’ trajectories. However, the majority of research and scientific knowledge in this field is based on cross-sectional studies. The translational gap in applying this knowledge to inform clinical management can only be bridged by research that systematically examines follow-up. In this study, we present findings from a novel longitudinal study of the long-term recovery trajectory of 39 patients, repeatedly assessed using the Coma Recovery Scale-Revised once every 3 months for 2 years, generating 185 assessments. Despite the expected inter-patient variability, there was a statistically significant improvement in behaviour over time. Further, improvements began approximately 22 months after injury. Individual variation in the trajectory of recovery was influenced by initial diagnosis. Patients with an initial diagnosis of unresponsive wakefulness state, who progressed to the minimally conscious state, did so at a median of 485 days following onset—later than 12-month period after which current guidelines propose permanence. Although current guidelines are based on the expectation that patients with traumatic brain injury show potential for recovery over longer periods than those with non-traumatic injury, we did not observe any differences between trajectories in these two subgroups. However, age was a significant predictor, with younger patients showing more promising recovery. Also, progressive increases in arousal contributed exponentially to improvements in behavioural awareness, especially in minimally conscious patients. These findings highlight the importance of indexing arousal when measuring awareness, and the potential for interventions to regulate arousal to aid long-term behavioural recovery in disorders of consciousness.

## Teaser

Patients with disorder of consciousness showed longitudinal improvements in behaviour consistent with a pattern of late recovery. Variability in recovery was predicted by initial diagnosis and age. It was progressively influenced by arousal, highlighting the importance of measuring arousal and the potential for arousal interventions to aid recovery.

## Graphical Abstract

**Graphical Abstract:**
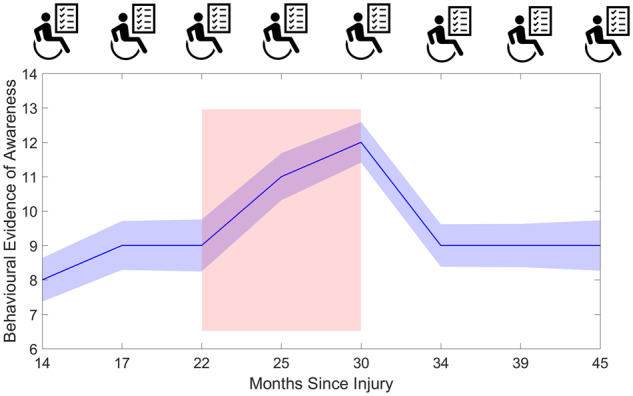

## Introduction

The degree of variability amongst patients with prolonged disorders of consciousness (pDOC) makes accurate diagnosis challenging (Royal College of Physicians, 2013). Prognostication is similarly difficult, as patients are often not followed up regularly. In large part, this is because of fragmentation of care over the patient journey: they are often transferred to specialist rehabilitation centres or to the family home following acute care, with incomplete records of their recovery history and outcomes. Consequently, the majority of research in this field has focused on cross-sectional research with convenience samples that do not inform on the natural history of recovery. Moreover, the degree of variability surrounding many existing prognostic indicators makes accurate prognostication challenging. To this end, the pDOC practice guidelines highlight the need for more systematic longitudinal research to relate clinical presentation to outcomes (Royal College of Physicians, 2013; Giacino *et al.*, 2018).

To date, research using systematic behavioural assessment has either followed patients for up to 12 months (Bagnato *et al.*, 2017), or with relatively long gaps, e.g. every 12 months for 4 (Katz *et al.*, 2009) or 5 years (Luaute *et al.*, 2010), or unevenly, e.g. at 1, 2 and 5 years post-injury (Nakase-Richardson *et al.*, 2012). Hence, this research has potentially missed when changes in consciousness occur. This is particularly problematic for unresponsive wakefulness state (UWS)/vegetative state patients, as detecting and predicting recovery to minimal consciousness (MCS) is important to inform treatment plans. Current UK clinical guidelines propose UWS permanence following 12 months with no change in consciousness after traumatic brain injury (TBI), and 6 months after non-traumatic injury (Royal College of Physicians, 2013). However, some patients have shown later recovery from UWS (Andrews, 1993; Childs and Mercer, 1996; Strauss *et al.*, 2000; Jennett, 2002; Faran *et al.*, 2006; Estraneo *et al.*, 2010; Kuehlmeyer *et al.*, 2013; Estraneo *et al.*, 2014; Steppacher *et al.*, 2014). To this end, the update to the clinical guidelines recommend that diagnoses of permanence should no longer be made in disorders of consciousness (DOC; Giacino *et al.*, 2018). These cases of late recovery highlight the challenge clinicians face in providing an accurate prognosis. Further, many reports reference symptom evolution in these patients referenced to the time of admission to the care facility, rather than the onset of the illness, limiting generalizability (Giacino *et al.*, 2018).

Patients’ presentation of behavioural responsiveness can also change dramatically with fluctuations of arousal (Wilson *et al.*, 1996; Giacino *et al.*, 2004; Wannez *et al.*, 2017). These fluctuations no doubt contribute to the high (40%) rate of misdiagnosis of conscious awareness in pDOC patients (Schnakers *et al.*, 2009). Consciousness is described as an interaction between arousal and awareness (Laureys, 2005), and the Coma Recovery Scale-Revised (CRS-R; Giacino *et al.*, 2004) is considered the most reliable and robust measure of consciousness for pDOC (Bodien *et al.*, 2016). Problematically, scores on the CRS-R can change across a week (Giacino *et al.*, 2004; Wannez *et al.*, 2017) or within a day (Candelieri *et al.*, 2011; Cortese *et al.*, 2015) due to patient fluctuations (Seel *et al.*, 2010). Whilst changes in arousal subscores naturally influence total CRS-R scores, the degree to which arousal modulates CRS-R scores has yet to be formally tested.

To address this gap in knowledge, we used the CRS-R (Giacino *et al.*, 2004; Bodien *et al.*, 2016) in a systematic longitudinal study to assess a group of patients at the bedside every 3 months across 2 years. Importantly, we chose to include both DOC patients both early and late stages of their behavioural trajectory. The first objective of this novel study design was to characterize the natural history of recovery, and emergence to higher states of consciousness, to identify the important predictors of CRS-R trajectories within and well beyond 12 months. Another objective was to investigate the effect of arousal fluctuations on patients’ longitudinal CRS-R trajectories, using the arousal subscale of the CRS-R. Importantly, we employed a valid systematic statistical approach [general linear mixed model (GLMM)], to investigate effects whilst controlling for other possible sources of variation of patient trajectories, which also reduced multiple testing.

## Materials and methods

### Ethics

This study was carried out in accordance with the recommendations of the UK National Health Service Research Ethics Committee for Cambridgeshire (reference: 16/EE/0006). Patients’ next-of-kin gave written informed consent or, in the absence of a suitable next-of-kin, the ward manager acted as a nominated consultee and provided written informed consent prior to enrolment in accordance with the UK Mental Capacity Act 2005 and Declaration of Helsinki.

### Participants

Patients were recruited from and assessed at two specialist neurological rehabilitation centres, where they received consistent and specialized care throughout the study. Two patients resided at one centre and the other 37 resided at the other centre. To be recruited, patients needed to be aged 16 years or older and clinically diagnosed with an apparent pDOC following any form of sudden onset, non-progressive brain injury. Patients must have been referred to or under review of a Consultant in rehabilitation medicine or Consultant Neurologist. Patients were excluded in the instance of pregnancy, if they were clinically unstable or, if they were diagnosed with a progressive neurological disease involving the brain or a serious mental health condition prior to their brain injury that has required active management by a psychiatrist. Patients who emerged from a DOC during or immediately prior to participation in the study were also excluded.

**Figure 1:**
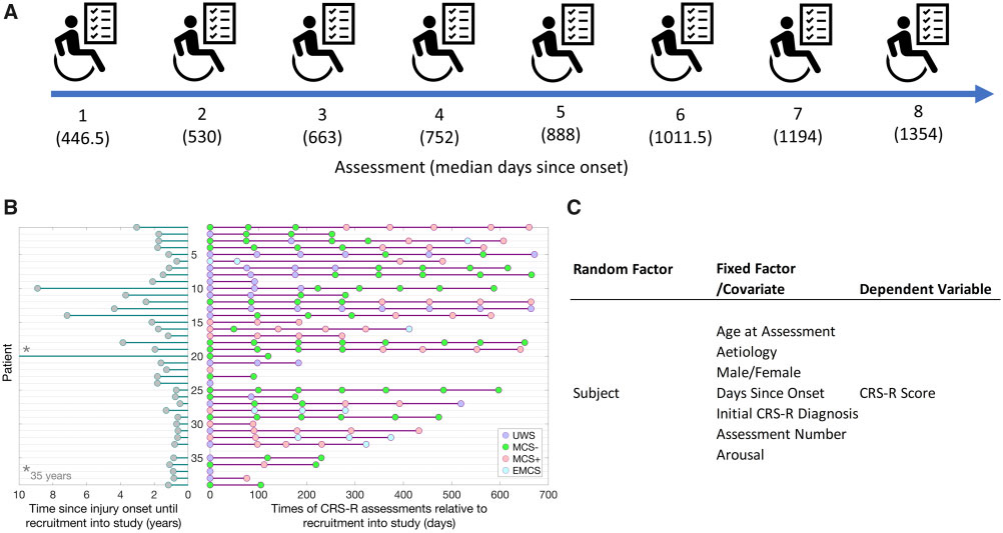
**Longitudinal design of the project and the statistical model.** (**A**) Illustration of the longitudinal design of the project. Patients were assessed at the bedside every 3 months with the CRS-R. Data collection began in June 2016 and was completed in June 2018. Patients were recruited at any point in the data collection period up until February 2018 to obtain a minimum of two assessments. (**B**) Figure illustrating, for each patient, time elapsed since injury onset at the point of recruitment (*left*), alongside the timeline of individual assessments and CRS-R diagnoses (*right*). Patients are ordered by time of recruitment into the study, and those recruited later had fewer assessments at the end of the 2-year study period. (**C**) Table of the independent random and fixed factors included in the GLMM.

Patients were assessed by neurologists throughout the duration of the study. A convenience sample of 40 patients were recruited; however, one patient passed away prior to the first scheduled assessment. The analyses presented here are based on the remaining 39 patients. Of these, 16 had an initial CRS-R diagnosis of UWS, 15 were MCS− (minimally conscious minus; no evidence of command following) and 7 MCS+ (minimally conscious plus; evidence of command following) and 1 EMCS (emerged from a minimally conscious state). Eighteen patients had an aetiology of TBI, with the remaining 22 had an anoxic (14), stroke (5) or other (2) injury. The patients (22 male, 17 female) were aged 19–75 years (*M* = 42.85, SD = 15.75) and were 174–12,880 days post-ictus (*M* = 1018.64, SD = 2056.77). This sample of prolonged DOC patients allows for the systematic assessment of the later history of recovery, beyond 12 months post-injury, to identify predictors of more longer-term outcomes for these patients. The number of patients declined across assessment number (*N* = 39, 36, 29, 23, 19, 15, 13, 10 for Assessments 1–8, respectively) due to attrition or late recruitment into the study (see Fig. 1B). All time points cited in this article are referenced to the date of ictus, rather than date of admission to their care facility.

### Design

The same researcher (C.A.B.) assessed the patients every 3 months at the bedside using the CRS-R to determine changes in behaviour. The researcher was formally trained on CRS-R administration prior to commencement of the study. Patients’ CRS-R diagnoses from the assessments reported here were anecdotally corroborated by independent CRS-R assessments conducted by a rehabilitation specialist (J.A.) as a part of clinical practice. The researcher was blind to these independent assessments conducted by the specialist. In total, data from 185 assessments (see Supplementary Table 1) from the 39 patients were included in the analysis (see Fig. 1).

### Coma Recovery Scale-Revised

The CRS-R is a 23-item scale behavioural assessment of awareness for pDOC (Giacino *et al.*, 2004). The scale is split into subscales that measure the auditory, visual, motor, oromotor/verbal, communication and arousal levels of the patient. Some items are considered to be signs of consciousness, with the most complex items indicating EMCS. The CRS-R was administered by the same trained neuropsychologist (C.A.B.) with each patient once at each time point. If possible, the patient was assessed upright in the chair. If this was not possible, patients were assessed at the bedside with the bed elevated to an upright sitting position. When it was required, the arousal intervention of applying deep pressure as per the CRS-R guidelines was administered prior to and, if necessary, throughout the duration of the examination to ensure the patient achieved peak possible arousal during the assessment.

### Statistical analysis

All 185 behavioural and demographic measures collected from patients and assessments were entered into a full GLMM with a Poisson distribution (suitable for the discrete, fixed CRS-R scale) and robust covariance (to account for outliers). A random factor of subject was included to account for within-subject variability and the different number of observations per subject. See Fig. 1C for details of the model design. SPSS syntax was used to fit the GLMM.

### Data availability

Data from the 185 assessments used in the presented analysis is available in the Supplementary material.

## Results

### Longitudinal trajectories of Coma Recovery Scale-Revised scores

**Figure 2:**
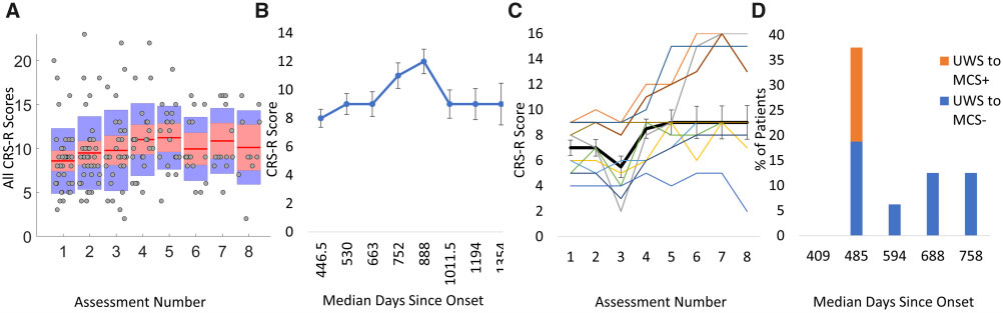
**Patients’ behavioural recovery over time.** (**A**) Boxplots showing the CRS-R scores of the patients across assessment. Red lines represent the medians and shaded areas represent the inter-quartile ranges. Individual scores are indicated as grey dots. (**B**) Illustration of the mean CRS-R scores plotted against the median days since onset (three patients’ data were removed from this graph as observations were >2 × SDs above the group mean). Error bars represent the standard errors for the group. (**C**) Trajectory of mean CRS-R scores (black line) of a subgroup of 10 patients who all had eight assessments. Standard error bars represent the standard error of the mean. Trajectories of individual patients in the group are plotted as thin coloured lines. (**D**) Stacked bar chart showing the proportion of UWS patients that changed diagnosis against the median days since injury onset at the time of the change.

There was a main effect of assessment number (*F*(7,132) = 2.88, *P* < 0.01), highlighting that, overall, CRS-R scores improved with assessment number (see Fig. 2A and B). Pairwise contrasts (Bonferroni corrected) indicated that whilst there was no difference among assessments 1, 2 or 3, there was a significant change in CRS-R from assessment 3 to 4 [*t*(132) = −5.16, *P* < 0.001, contrast estimate (CE) = −3.06], 5 to 6 [*t*(132) = −3.18, *P* = 0.03, CE = −1.31], 6 to 7 [*t*(132) = −4.727, *P* < 0.001, CE = −2.48] and 7 to 8 [*t*(132) = −10.93, *P* < 0.001, CE = −4.78].

To verify the pattern of recovery remained in the 10 patients with all eight assessments, the model was fitted to just that subgroup (Fig. 2C). The main effect of assessment number remained with assessments 1–3 differing from assessments 4–7 (all contrasts = *P* < 0.05 Bonferroni corrected).

### Days since onset

The model indicated a significant main effect of days since onset of injury—*F*(1,132) = 15.20, *P* < 0.001)—indicating that this was a significant predictor of CRS-R scores. As Fig. 2B shows the upward shift in CRS-R scores occurs after 663 days (approximately 22 months) since onset. This is in line with a meta-analysis of pDOC case studies, which indicated that, in the natural history of recovery, diagnosis can improve 22–25 months post-injury (Giacino *et al.*, 2018). An interaction between assessment number and days since onset—*F*(7,132) = 4.21, *P* = <0.001—indicated that days since the onset of injury could explain the observed change in CRS-R scores over time.

Figure 2D shows the proportion of patients with an initial UWS diagnosis that recovered to a MCS (−/+) diagnosis, as a function of median days since onset. Of the 16 patients with an initial UWS diagnosis, 11 improved to MCS (68.75%) within the 2-year period of assessments. The majority improved at assessment 2 (*N* = 6)—with three patients scoring a CRS-R diagnosis of MCS+—at a median of 485 days since injury. For patients with an initial MCS− diagnosis (*N* = 15), only 6 (40%) showed an improvement to MCS+ that was maintained in subsequent assessments. For those that did change, half (*N* = 3) showed this change at assessment 5—a median of 1167 days since onset. Two patients improved to MCS+ before then, one at assessment 3 and one at assessment 4 (median days since onset = 992 and 1084, respectively), whilst the other showed improved CRS-R diagnosis on assessment 6 (1152 days since onset). These changes in CRS-R diagnosis occur much later for patients with an initial MCS diagnosis than those with an initial UWS diagnosis. One patient with an initial MCS− diagnosis who progressed to MCS+ went on to EMCS on assessment 6 (1051 days since onset) evidenced by functional and accurate communication. Two patients with an initial MCS+ diagnosis progressed to EMCS, one at assessment 2 (564 days since onset) and one at assessment 3 (407 days post-ictus), evidence by functional communication for one and functional object use for the other.

### Late recovery from unresponsive wakefulness state to minimal consciousness

To determine statistically whether our sample of UWS patients showed evidence of late recovery (beyond the 12 month period suggested to indicate permanence; Royal College of Physicians, 2013), we ran the same GLMM on *just* those patients with an initial UWS diagnosis (*N* = 16 patients, 75 assessments) on only the data collected after 12 months post-ictus. Even in this subset of data, there was a main effect of assessment number, *F*(7,45) = 17.80, *P* < 0.001, with significant improvement in CRS-R scores seen at assessment 5 (assessments 1–4 differed from assessment 5, all *P* < 0.001 Bonferroni corrected) with a slight plateau in scores from assessments 6–8 (only assessment 1 significantly different from assessment 6, and assessments 1 and 2 differed from assessments 7 and 8 all *P* < 0.05 Bonferroni corrected). In sum, the analysis indicated improvement in CRS-R scores well beyond 12 months (assessment 1 median days post-ictus = 646, range = 409–3251) with significant changes in behaviour at assessment 5 (median days post-ictus = 764.5, range = 566–3560).

In line with previous reports (Giacino and Kalmar, 1997; Bagnato *et al.*, 2017), 10 of the 11 (91%) patients who changed from UWS to MCS showed the first signs of consciousness on the visual subscale. The other patient (UWS to MCS+) showed the first sign of consciousness on the auditory scale with reproducible movement to command (no evidence of visual pursuit). On the visual subscale, consciousness was first evidenced with visual fixation for two patients with the remaining eight showing visual pursuit. One of the patients who progressed from UWS to MCS+ directly went on to EMCS at Assessment 5 (610 days since injury), as evidenced by functional and accurate communication.

### Initial Coma Recovery Scale-Revised diagnosis

**Figure 3:**
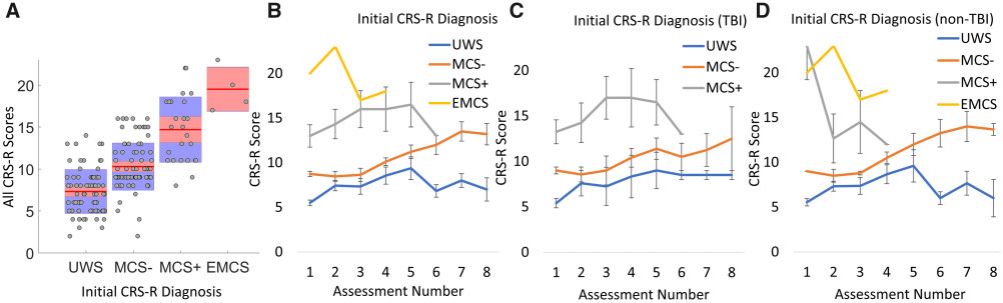
**Initial diagnosis as a predictor of recovery.** (**A**) Boxplot of all 185 CRS-R scores collected from all patients, grouped by initial diagnosis. Pairwise Bonferroni contrasts showed no difference between MCS patients but statistical differences between UWS and both the MCS− and MCS+ groups. (**B**) Mean CRS-R scores for patients grouped by initial diagnosis at each assessment. (**C**) Mean CRS-R scores at each assessment for patients with a TBI aetiology grouped by initial diagnosis. (**D**) Mean CRS-R scores at each assessment for patients with a non-TBI aetiology grouped by initial diagnosis. Standard error bars represent the standard errors of the means for all plots.

**Figure 4:**
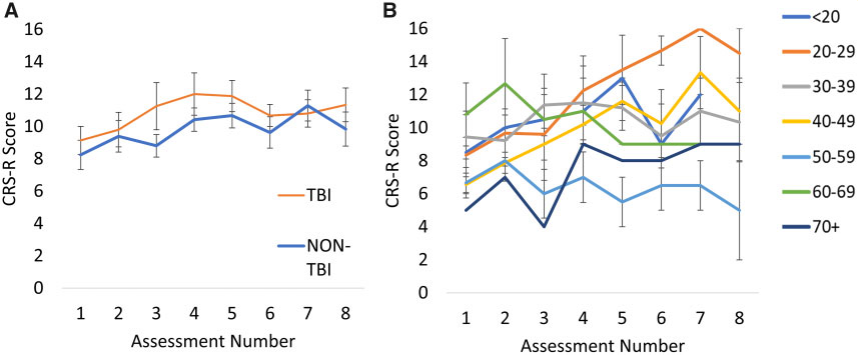
**CRS-R scores grouped by aetiology and age.** (**A**) Mean CRS-R scores for patients grouped by aetiology. Standard error bars represent standard errors of the mean. (**B**) Illustration of trajectories for patients grouped by age. Younger patients, particularly those 20–29 years show a more promising trajectory with higher CRS-R scores on later assessments than older patients. Standard error bars represent standard errors of the mean.

There was a main effect of initial CRS-R diagnosis (based on the first assessment) on CRS-R scores (Fig. 3A):*F*(2,132) = 7.60, *P* < 0.01. Pairwise Bonferroni contrasts indicated overall differences between patients diagnosed as UWS and MCS− [*t*(132) = −4.41, *P* < 0.001, CE = −1.35] and UWS and MCS+ [*t*(132) = −3.38, *P* < 0.01, CE = −2.15]. There was no statistical difference between the MCS− and MCS+ patients (see Fig. 4) and no difference between any of the groups of patients and the EMCS patient, likely due to a lack of statistical power (*N* = 1 patient, four assessments). A two-way interaction between initial diagnosis and assessment number (*F*(11,132) = 9.89, *P* < 0.001) suggested that patients’ initial diagnoses played a significant part in their consequent trajectory of recovery (see Fig. 3B), irrespective of aetiology (Fig. 3C and D).

### Age, gender and aetiology

Unlike previous literature (Jennett, 2002; Estraneo *et al.*, 2010), there was no main effect of aetiology on CRS-R scores, and no two-way interaction between aetiology and assessment number in our data (*P* > 0.05, see Fig. 4A). Overall, 10 of the 18 TBI patients showed an improvement in CRS-R diagnosis (55%). Similarly, 10 of the 21 non-TBI patients (47%) also showed improvement on CRS-R diagnosis across the 2-year period. It is possible that the non-significant effect of aetiology is due to many of the assessments occurring at more chronic stages, somewhat later than previous literature that has shown aetiology as important to predict outcome. To investigate this, the interaction between aetiology and days since onset was assessed. There was no significant interaction *F*(1,145) = 0.80, *P* = 0.37 between aetiology and day since onset indicating that CRS-R scores were similar between TBI and non-TBI subgroups regardless of the time since injury. Aetiology has been shown to predict long-term outcome for UWS patients but not MCS patients (Steppacher *et al.*, 2014). In our sample of UWS patients, there was no main effect of aetiology but there was a significant interaction between aetiology and assessment number, *F*(7,47) = 17.78, *P* < 0.001; however, the pairwise contrasts did not survive Bonferroni correction likely due to insufficient power. Although, it is worth noting the two TBI patients initially in a UWS state that recovered to a MCS+, emerged within the first 12 months post-ictus.

There was no main effect of age at first assessment (*P* > 0.05) but there was a two-way interaction between age and assessment number, *F*(7,132) = 3.19, *P* < 0.001). Although patients start with similar CRS-R scores (see Fig. 4B), younger patients—particularly those aged 20–29—show a more promising rate of recovery with increases in CRS-R scores continuing onto later assessments. Age as a covariate of recovery has also shown to depend on initial diagnosis (Steppacher *et al.*, 2014) with age only having an effect on outcome for UWS patients. In our sample of UWS patients, there was no main effect of age but age did interact with assessment number, *F*(7,54) = 67.71, *P* < 0.001, consistent with previous findings.

There was a main effect of gender—*F*(1,132) = 9.09, *P* < 0.01—with males (*M* = 10.28, SD = 4.07) having a higher CRS-R scores than females (9.48, SD = 3.98) overall *t*(132) = −3.05, *P* < 0.01, CE = −1.17. However, there was no two-way interaction of gender by assessment number (*P* > 0.05), indicating a similar trajectory for both males and females.

### Arousal

**Figure 5:**
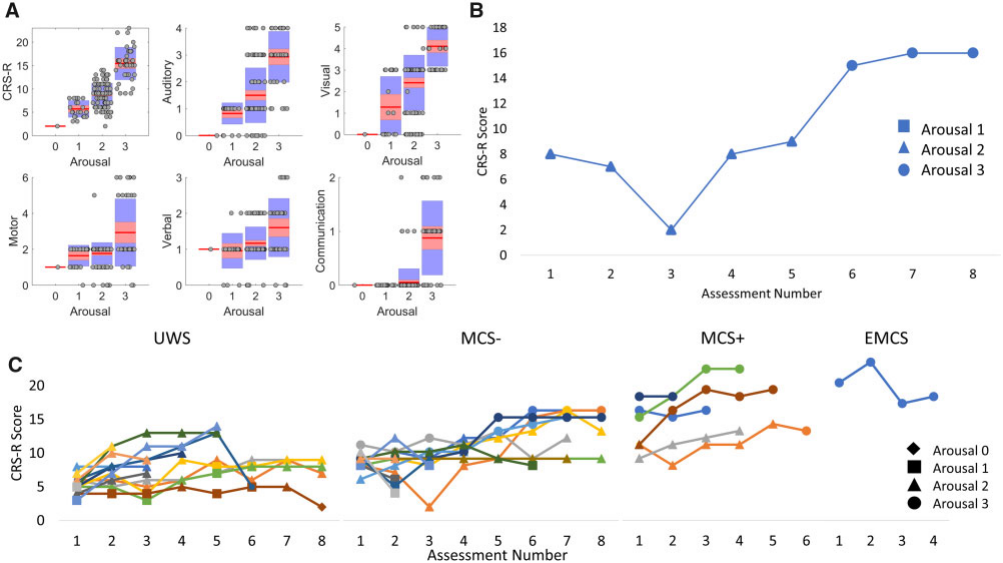
**Arousal as an important predictor of recovery.** (**A**) Boxplots illustrating the total CRS-R scores and the subscale scores associated with each categorical score on the arousal subscale. The plots demonstrate that higher scores on all the subscales typically occur at highest arousal scores. (**B**) Example of an (initially) MCS− patient that shows higher CRS-R scores at later assessments (6–8) that coincides with higher scores on the arousal subscale. (**C**) Individual CRS-R score trajectories across assessments for each patient grouped by Initial diagnosis. Individual patients’ trajectories are represented by different coloured lines. For most patients, there are clearly higher CRS-R scores when the arousal subscale score is also higher. CRS-R scores are typically higher for later assessments and those later CRS-R scores are higher for patients with an MCS or EMCS initial diagnosis.

The arousal subscale of the CRS-R score was used as a quasi-independent categorical measure of wakefulness. It was expected that arousal scores would affect the other subscale scores. In line with this, there was a main effect of arousal [*F*(3,132) = 188.79, *P* < 0.001], with all possible pairwise contrasts statistically significant (*P* < 0.001 Bonferroni corrected). Figure 5A shows that increases in the arousal subscale led to greater CRS-R scores, above and beyond the amount of increase in the arousal subscore itself.

There was also a two-way interaction between arousal and assessment number, *F*(11,132) = 2.86, *P* < 0.01, across all patients indicating that CRS-R score trajectories depend on arousal (all possible pairwise contrasts *P* < 0.01 Bonferroni corrected). For many patients, higher arousal scores coincided with higher CRS-R scores at later assessments. As an exemplar, Fig. 5B shows an example of an MCS− patient who demonstrates a change in the slope of CRS-R scores from assessments 1–4 to assessments 5–8, reflecting a shift in CRS-R recovery coinciding with increased arousal.

In patients with an initial diagnosis of UWS, there was a significant interaction between arousal and assessment number, *F*(4,57) = 5.26, *P* < 0.01. CRS-R scores differed significantly depending on the arousal scores for all assessments except for assessments 2 and 6 (Bonferroni adjusted *P* > 0.05). In patients with an initial MCS (−/+) diagnosis, there was again a significant interaction between arousal and assessment number, *F*(10,82) = 4.33, *P* < 0.001. For these patients, CRS-R score depended on arousal at every assessment (*P* < 0.05 Bonferroni corrected). Higher arousal scores seem to occur in later assessments for the UWS and MCS− groups (see Fig. 5C). However, high arousal seems to occur at both early and late assessments for the MCS+ group and the single EMCS patient.

## Discussion

### Time since injury predicts recovery

Our findings from this longitudinal study have several important clinical and ethical implications. Similar to research of short-term outcomes (Whyte *et al.*, 2009), time since injury was an important predictor of CRS-R trajectory across the 2-year period. Our cohort of prolonged DOC patients began to show improvements in CRS-R scores after assessment 3—22 months post-onset. It is important to note that these signs of improvement are somewhat later than many of those seen in studies of other, less chronic, samples and might reflect the particular nature of our sample. Nonetheless, these late signs of recovery have particular relevance for UWS. For the UWS patients who improved to MCS, the median days post-onset was 445 days—much later than the 12-month timeline current guidelines propose for diagnosing UWS permanence (Royal College of Physicians, 2013). This finding adds to the weight of evidence from several studies demonstrating recovery beyond 12 months post-onset (Andrews, 1993; Childs and Mercer, 1996; Strauss *et al.*, 2000; Faran *et al.*, 2006; Estraneo *et al.*, 2010; Kuehlmeyer *et al.*, 2013; Estraneo *et al.*, 2014; Steppacher *et al.*, 2014). A recent review reported cases of UWS patients emerging to MCS or full recovery up to 6 years post-TBI (Kuehlmeyer *et al.*, 2013). Together these findings suggest that, in line with the recent update to practice guidelines (Giacino *et al.*, 2018), a diagnosis of permanence, if made at all, should not be within the first 24 months post-ictus.

Inaccurate diagnosis of UWS permanence could lead to patients prematurely being treated palliatively, focussing on management rather than rehabilitation, leading to poorer outcomes. Moreover, inaccurate diagnosis of UWS permanence could lead to a nihilistic approach to care, reducing potential for improvement (Kuehlmeyer *et al.*, 2013). The low expectation of recovery might bias family decisions regarding life-sustaining treatment towards termination (Kuehlmeyer *et al.*, 2013). These issues highlight the importance of longitudinal research to arm clinicians with more evidence to provide an accurate prognosis following severe brain injury. Conversely, the timeframe and extent of recovery and influence of age can critically inform discussions with families, providing a framework for best interests decisions taking into account patients’ age, comorbidities and individual choice.

### The importance of diagnostic accuracy

Rates of misdiagnosis in pDOC are recorded at approximately 40% (Schnakers *et al.*, 2009). The substantial range of complex comorbid difficulties and disorders patients often present with, as well as variability in medications and available treatments, contributes to this diagnostic challenge. Problematically, the findings here indicate that misdiagnosis could also lead to incorrect prognostication. MCS patients showed a typically consistent steady increase in CRS-R scores over time whilst UWS patients showed an initial increase that plateaued 18 months after injury. This plateau is likely due to high attrition with those surviving having reached peak behavioural responsiveness by this time. This finding is consistent with research that shows a greater odd of recovery for MCS patients (Giacino and Kalmar, 1997; Strauss *et al.*, 2000; Lammi *et al.*, 2005; Voss *et al.*, 2006; Giacino *et al.*, 2018).

The majority (4/5) of the UWS patients who did not recover to MCS within 2 years had a non-traumatic aetiology. This is consistent with the research that has found that typically poorer outcomes for patients with a non-traumatic injury (Giacino and Kalmar, 1997; Jennett, 2002; Giacino, 2004). Despite this, aetiology did not significantly predict CRS-R score trajectories in this sample. One possibility is that whilst aetiology may not be a significant predictor of outcome for more chronic patients, such as those reported here, it is still an important predictor of recovery in earlier stages post-injury. This could explain the inconsistency of our findings to previous research investigating predictors of recovery in less chronic DOC patients. Although there was no significant interaction between aetiology and day since onset in our sample, it could be that the majority of the assessments here occurred at much later stages post-injury, by which point aetiology no longer has predictive power. Another possibility is that the effect of aetiology is reduced due to the inclusion of initial diagnosis as a factor in the model. In line with previous literature (Steppacher *et al.*, 2014), aetiology did interact with assessment number on CRS-R scores for the UWS patients. The model also identified age as an important predictor of CRS-R trajectories. This finding is in concordance with the recent update to practice guidelines (Giacino *et al.*, 2018), and previous research has demonstrated that younger patients typically have a better outcome (Braakman *et al.*, 1988; Estraneo *et al.*, 2010).

### First signs of consciousness

In line with previous research, progression from the UWS to the MCS− in our data was first seen with visual pursuit or fixation (Seel *et al.*, 2010; Bagnato *et al.*, 2017). In patients that progressed to MCS+, command following was often evidenced with accurate object selection using eye-related movements. Likewise, during progression to EMCS, the majority (3/4) evidenced awareness via accurate communication—typically using eye blinks or selecting ‘Yes/No’ using gaze. These observations are not surprising, given these patients often have substantial motor deficits, making progression on the motor function scale somewhat challenging and unreliable. Further, vocal responses are challenging for patients with tracheostomy. Instead, movement of the eyes to communicate or demonstrate awareness seems to be more achievable for patients at all stages of recovery from severe brain injury.

### The importance of arousal for behavioural assessments of consciousness

Our statistical analysis shows that arousal is an important factor contributing to CRS-R total scores. Increases in the arousal subscale by just one point increased CRS-R total scores exponentially, by engendering increases in the other subscales. This validates the need for arousal interventions, such as that in the CRS-R guidelines (Giacino *et al.*, 2004), to ensure the patient is at peak possible arousal prior to administering behavioural assessments of awareness. It is worth noting that not all behavioural assessments include an arousal intervention (e.g. the Wessex Head Injury Matrix; Shiel *et al.*, 2000). The findings here suggest that such measures might produce an inaccurate representation of awareness state due to lower than optimal arousal levels.

CRS-R trajectories depended on arousal scores at every assessment with higher arousal scores associated with higher CRS-R scores. For patients with an initial UWS diagnosis, none demonstrated a level of arousal >2 even after emerging to MCS. This could be because the increase in CRS-R scores from an arousal score of 2–3 is considerable, and might only be achieved by those with an initial MCS diagnosis and a greater potential for recovery. For the MCS patients, those with high arousal earlier had higher CRS-R scores on later assessments. This is consistent with a report that indicated that the sequence of behavioural recovery began with arousal and led onto more complex signs of cognition (Shiel *et al.*, 2000). Arousal then, may have some prognostic value, predicting the likelihood of increases in overall behavioural responsiveness over time.

Moreover, interventions to increase arousal could have therapeutic benefits. Pharmacological approaches to increase arousal levels, such as the use of zolpidem and amantadine, have been shown to increase behavioural awareness in pDOC patients (Whyte and Myers, 2009; Giacino *et al.*, 2012; Whyte *et al.*, 2014). Further, the use of electrical stimulation such as anodal transcranial direct current stimulation (tDCS)—thought to increase cortical excitability (Thibaut and Laureys, 2015)—has shown to improve behavioural responsiveness in these patients, particularly MCS (Thibaut *et al.*, 2014; Thibaut and Laureys, 2015; Thibaut *et al.*, 2017). The effects of tDCS have been considered to be the result of increased frontal cortical excitability interacting with regions in the brainstem, such as the striatum and thalamus, involved in modulating arousal—an account called the mesocircuit model (Thibaut and Laureys, 2015). In contrast, pharmacological interventions are thought to target brainstem regions directly. Whether any of these stimulation methods have long-term benefits is yet to be formally assessed, although one study showed some promise with repeated assessments (*N* = 5) of anodal frontal tDCS showing improvements in some MCS patients that lasted a week later (Thibaut *et al.*, 2017). Our findings suggest that such methods to increase arousal should be included in rehabilitation strategies to achieve better outcomes, particularly for MCS patients.

In addition, the powerful effect of arousal on behaviour suggests that arousal is likely to have a similarly important effect of neuroimaging assessments of awareness. Recent advances in clinical neuroimaging have highlighted their complementarity to behavioural assessments, with the potential to improve diagnosis and prognosis. Arousal fluctuations then, need to be factored in to the development of neuroimaging tools for detecting awareness in pDOC.

### Limitations

Previous research has shown that at least four CRS-R assessments are required to achieve an accurate score/diagnosis (Wannez *et al.*, 2017). Here, the CRS-R was administered once every 3 months, as multiple assessments at each time point was not feasible, either due to time restrictions or patient tolerance. Although the best care was taken to ensure peak arousal, there is a possibility that the patient trajectories presented here are nevertheless affected by arousal fluctuations. To account for this, the arousal subscale was included as a factor in the model, controlling for such fluctuations as best as possible.

There are differing numbers of observations between patients due to attrition and because patients were recruited at any time point up until 3 months before the end of data collection. To account for this, a random factor of Subject was included in the model. Further, the GLMM used a robust estimation method to account for outliers in the main analyses. Nevertheless, given that only one patient had an initial diagnosis of EMCS, results that compare this patient to other subgroups should be interpreted with caution.

All the patients in our sample resided in specialist centres with access to rehabilitation services that patients in other contexts may not have access to. Likewise, the majority of patients were recruited from one neurological centre. Further studies involving patients from multiple centres are required to better characterize the role of the rehabilitation context on patient trajectories.

Finally, an important limitation of this study was the use of a single examiner who collected the CRS-R scores reported here, and the consequent potential for undetected measurement error and potential variance. Although the relatively modest scale of the study necessitated this, future larger-scale studies might aim to employ multiple examiners and establish inter-rater reliability of longitudinal bedside measurements.

## Conclusions

The longitudinal research project described here demonstrates that pDOC patients do show long-term behavioural improvements post-injury, extending over 2 years and beyond. Whilst MCS patients show a more promising and continuing trajectory of recovery, UWS patients showed some initial improvement, and many do progress to a MCS beyond 12 months since injury. Our findings have shown that several factors including initial diagnosis and age need to be considered when making a clinical prognosis. We have also shown that arousal variation is an important predictor of trajectories. Moreover, these arousal fluctuations have an important influence on the behavioural assessment of consciousness at the bedside. This highlights that, like the CRS-R, arousal interventions should be included in the administration of systematic behavioural assessments. Arousal could also influence neuroimaging assessments of consciousness and arousal interventions could have long-term therapeutic benefits.

## Supplementary material

Supplementary material is available at *Brain Communications* online.

## Supplementary Material

fcz017_Supplementary_Data

## Abbreviations

CRS-R =: Coma Recovery Scale-Revised
DOC =: disorders of consciousness
GLMM =: general linear mixed model
EMCS =: emerged from minimal consciousness
MCS =: minimal consciousness
pDOC =: prolonged disorders of consciousness
TBI =: traumatic brain injury
tDCS =: transcranial direct current stimulation
UWS =: unresponsive wakefulness state
